# Supplementary material for: Dietary Patterns Related to Triglyceride and High-Density Lipoprotein Cholesterol and the Incidence of Type 2 Diabetes in Korean Men and Women
Source: Nutrients. 2018 Dec 20;11(1):8. doi: 10.3390/nu11010008 (PMC6357028; doi:10.3390/nu11010008)
Supplement: Supplementary file 1 [file nutrients-11-00008-s001.pdf]

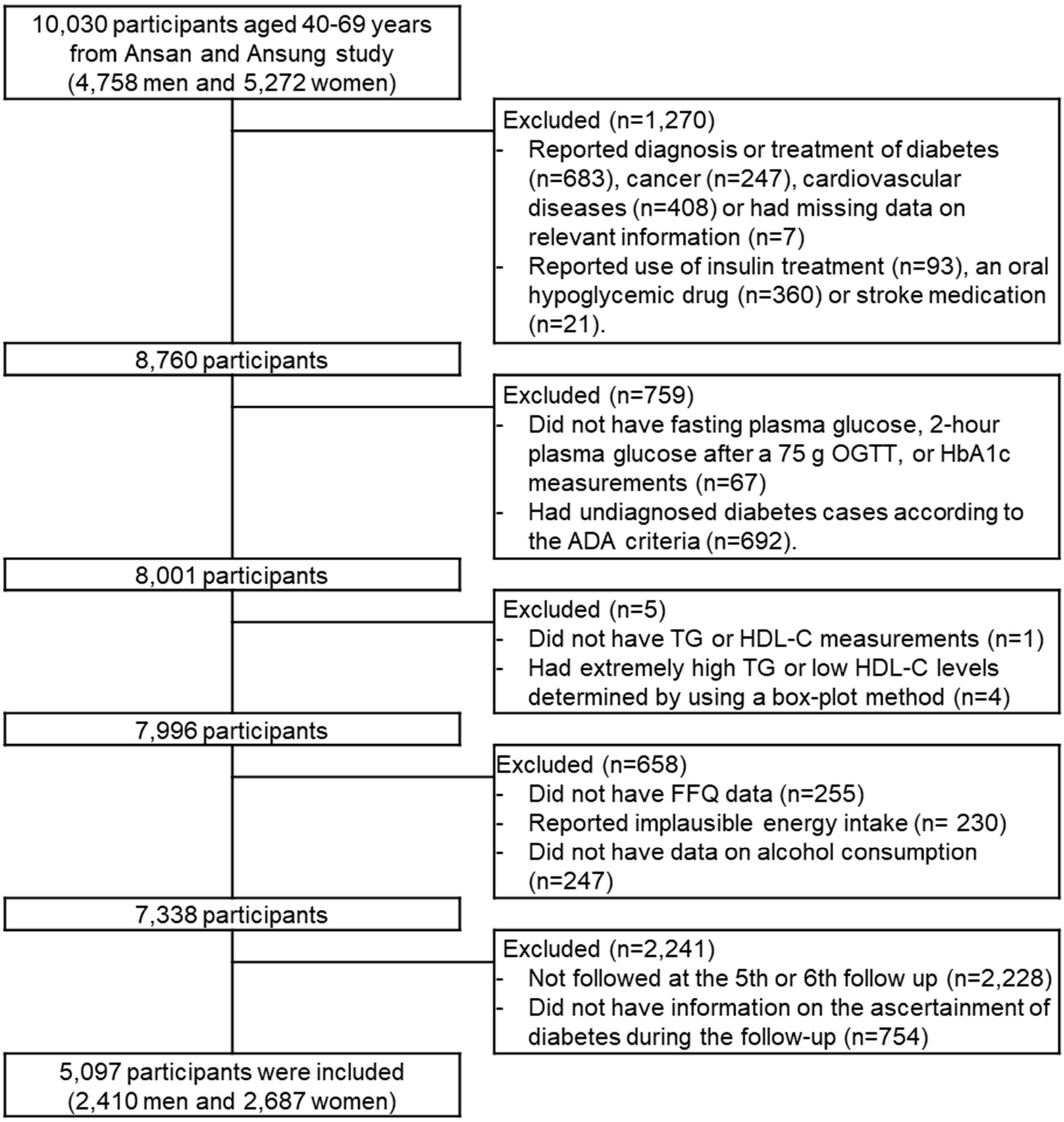

Figure S1. Flow diagram of inclusion for study participants. Abbreviations: OGTT, oral glucose tolerance test; HbA1c, hemoglobin A1c; ADA, American Diabetes Association; TG, triglyceride; HDL–C, high–density lipoprotein cholesterol; FFQ, food frequency questionnaire.

Table S1. Odds ratios (ORs) and 95% confidence intervals (CIs) of incident type 2 diabetes according to the quintiles of TG/HDL-C ratio-related dietary pattern after excluding incident cases during 2 years of' follow up.

	Quintiles of TG/HDL-C ratio-related dietary pattern scores					
	**Quintile1**	**Quintile2**	**Quintile3**	**Quintile4**	**Quintile5**	***p* for trend**
Men (*n* = 2298)						
Case/noncase	81 / 378	86 / 374	85 / 375	86 / 374	110 / 349	
Unadjusted model	Reference	1.07 (0.77–1.50)	1.06 (0.76–1.48)	1.07 (0.77–1.50)	1.47 (1.07–2.03)	0.017
Age-adjusted model	Reference	1.08 (0.77–1.51)	1.06 (0.75–1.48)	1.07 (0.77–1.50)	1.46 (1.06–2.01)	0.021
Multivariate adjusted model1 [a]	Reference	1.11 (0.79–1.57)	1.09 (0.77–1.54)	1.11 (0.79–1.56)	1.56 (1.12–2.18)	0.010
Multivariate adjusted model2 [b]	Reference	1.10 (0.78–1.56)	1.06 (0.74–1.50)	1.04 (0.74–1.47)	1.51 (1.08–2.11)	0.023
Women (*n* = 2606)						
Case/noncase	75 / 447	64 / 452	99 / 424	96 / 455	94 / 400	
Unadjusted model	Reference	0.84 (0.59–1.21)	1.39 (1.00–1.93)	1.26 (0.91–1.75)	1.40 (1.01–1.95)	0.007
Age–adjusted model	Reference	0.84 (0.59–1.20)	1.39 (1.00–1.93)	1.21 (0.87–1.68)	1.29 (0.92–1.80)	0.025
Multivariate adjusted model1 [c]	Reference	0.82 (0.57–1.19)	1.38 (0.99–1.94)	1.17 (0.82–1.67)	1.28 (0.90–1.84)	0.039
Multivariate adjusted model2 [b]	Reference	0.79 (0.54–1.14)	1.31 (0.93–1.84)	1.07 (0.74–1.53)	1.15 (0.80–1.65)	0.155

Abbreviations: TG, triglyceride; HDL–C, high–density lipoprotein cholesterol. [a] Adjusted for age (continuous, years), living area (Ansan and Ansung), energy intake (continuous, kcal/day), pack–years of smoking (0, >0 and <15, 15–<30, and 30≤ pack–years), alcohol consumption (0, >0 and <5, 5–<15, 15–<30 and 30≤ g/day), family history of diabetes (yes, no), hypertension or hyperlipidemia at baseline (yes, no), and physical activity (continuous, metabolic equivalents–hours/week). [b] Further adjusted for BMI (continuous, kg/m^2) in addition to the variables included in Model 1. [c] Adjusted for age (years), living area (Ansan and Ansung), energy intake (continuous, kcal/day), menopausal status (pre and postmenopausal status), smoking status (ever and never), alcohol consumption status (ever and never), family history of diabetes (yes, no), hypertension or hyperlipidemia at baseline (yes, no), and physical activity (continuous, metabolic equivalents–hours/week).
